# TOPK Promotes Microglia/Macrophage Polarization towards M2 Phenotype via Inhibition of HDAC1 and HDAC2 Activity after Transient Cerebral Ischemia

**DOI:** 10.14336/AD.2017.0328

**Published:** 2018-04-01

**Authors:** Ziping Han, Haiping Zhao, Zhen Tao, Rongliang Wang, Zhibin Fan, Yumin Luo, Yinghao Luo, Xunming Ji

**Affiliations:** ^1^Cerebrovascular Diseases Research Institute and Department of Neurology, Xuanwu Hospital of Capital Medical University, Beijing 100053, China.; ^2^Beijing Institute for Brain Disorders, Beijing 100053, China.; ^3^Beijing Geriatric Medical Research Center, Beijing 100053, China.

**Keywords:** T-LAK-cell-originated protein kinase, microglia/macrophage, polarization, ischemia-reperfusion, histone deacetylase

## Abstract

T-LAK-cell-originated protein kinase (TOPK) is a newly identified member of the mitogen-activated protein kinase family. Our previous study has showed that TOPK has neuroprotective effects against cerebral ischemia-reperfusion injury. Here, we investigated the involvement of TOPK in microglia/ macrophage M1/M2 polarization and the underlying epigenetic mechanism. The expression profiles, co-localization and *in vivo* interaction of TOPK, M1/M2 surface markers, and HDAC1/HDAC2 were detected after middle cerebral artery occlusion models (MCAO). We demonstrated that TOPK, the M2 surface markers CD206 and Arg1, p-HDAC1, and p-HDAC2 showed a similar pattern of in vivo expression over time after MCAO. TOPK co-localized with CD206, p-HDAC1, and p-HDAC2 positive cells, and was shown to bind to HDAC1 and HDAC2. *In vitro* study showed that TOPK overexpression in BV2 cells up-regulated CD206 and Arg1, and promoted the phosphorylation of HDAC1 and HDAC2. In addition, TOPK overexpression also prevented LPS plus IFN-γ-induced M1 transformation through reducing release of inflammatory factor of M1 phenotype TNF-α, IL-6 and IL-1β, and increasing TGF-β release and the mRNA levels of TGF-β and SOCS3, cytokine of M2 phenotype and its regulator. Moreover, the increased TNF-α induced by TOPK siRNA could be reversed by HDAC1/HDAC2 inhibitor, FK228. TOPK overexpression increased M2 marker expression *in vivo* concomitant with the amelioration of cerebral injury, neurological functions deficits, whereas TOPK silencing had the opposite effects, which were completely reversed by the FK228 and partially by the SAHA. These findings suggest that TOPK positively regulates microglia/macrophage M2 polarization by inhibiting HDAC1/HDAC2 activity, which may contribute to its neuroprotective effects against cerebral ischemia-reperfusion injury.

Stroke remains the leading cause of death and long-term disability in developed countries [1, 2]. Thrombolytic therapy using recombinant tissue plasminogen activator (rtPA) is the only valid treatment for acute ischemic stroke [3]. Thus, effective treatments for stroke are urgently needed. As the first line of defense against brain injuries, microglia/macrophages respond dynamically to cerebral ischemia-reperfusion injury, showing an early neuroprotective M2 phenotype, followed by a transition to a pro-inflammatory M1 phenotype [4, 5]. These dual and opposing roles of microglia/macrophage suggest that suppressing microglia/macrophage phenotype transition is a promising strategy for the treatment of stroke.

Histone deacetylases (HDACs) control transcription and regulate cellular fate in cerebral ischemia, and HDAC inhibitors (HDACi) are involved in microglia/ macrophage inflammatory responses [6]. The broad-spectrum HDACi trichostatin A (TSA) and suberanilohydroxamic acid (SAHA) strongly suppress LPS-induced expression of cytokine IL-6 and TNF-α in primary mouse microglia [7]. The HDACi Scriptaid promotes oligodendrocyte survival indirectly through a GSK3β/PTEN/PI3K/Akt axis-mediated phenotypic shift in microglia/macrophage, exerting neuroprotective effects [8]. However, broad-spectrum HDACi have undesired side effects, which makes selective HDAC inhibition preferable [9].

T-LAK-cell-originated protein kinase (TOPK) is a novel member of the mitogen-activated protein kinase kinase (MAPKK) family that acts on the JNK, p38, ERK, and PTEN/PI3K/Akt pathways [10]. Inhibition of TOPK disrupts neural progenitor self-renewal and proliferation [11]. Our previous study has showed that TOPK activation confers neuroprotection against focal cerebral ischemia-reperfusion injury through an antioxidative effect [12]. Moreover, TOPK activation mediated anti-inflammation has been proved to involve in remote limb ischemic postconditioning invoked protection against renal ischemia/reperfusion injury [13], its function in microglia/macrophage polarization during cerebral ischemia-reperfusion injury remains largely unexplored.

Since HDACs are involved in microglia/macrophage M1/M2 polarization [8], and HDAC1 and HDAC2 are most abundantly expressed by microglia/macrophage and significantly changed by LPS stimulation *in vitro* and *in vivo* [7], we hypothesized that TOPK could influence microglia/macrophage M1/M2 polarization by regulating HDAC1/HDAC2 and histone acetylation, resulting in neuroprotection against cerebral ischemia-reperfusion injury. The present study was designed to test this hypothesis and explored possible therapeutic targets for the treatment of ischemic stroke.

## MATERIALS AND METHODS

### Animals

Male C57Bl/6 mice weighing 20-25g were purchased from Vital River Laboratory Animal Technology Co. Ltd. All the animal experiments in this study were approved by the Institutional Animal Care and Use Committee of Capital Medical University. We used as few animals as possible and all efforts were made to minimize their suffering.

### Induction of transient focal ischemia

To induce transient focal cerebral ischemia, male C57/BL6 mice (22-23g) were anesthetized with enflurane (4% induction, 1.5% maintenance in O_2_ at 0.2 L/min, N_2_O at 0.4 L/min) and subjected to intraluminal occlusion of the right middle cerebral artery (MCAO) as described previously [14, 15]. In brief, a silicon rubber-coated monofilament (diameter: 0.21 ± 0.02 mm; Doccol, CA) was inserted into the right external carotid artery lumen and gently advanced into the internal carotid artery until slight resistance was felt. To ensure the occurrence of ischemia by MCAO, regional cerebral blood flow (rCBF) was monitored using laser Doppler flowmetry (PeriFlux System 5000, Perimed, Sweden) at a location 0.5 mm anterior and 5.0 mm lateral from bregma. The ipsilateral cerebral blood flow decreased to 15~25% of pre-ischemia baseline levels. The filament was left in place for 45 min and then withdrawn. Animals in the sham group underwent the same anesthesia and surgical procedures except MCAO. The rectal temperature was maintained at 37.0 ± 0.5°C during and after the MCAO surgery via a temperature-regulated heating pad (CMA 150; Carnegie Medicin, AB, Stockholm, Sweden). After recovering from anesthesia, all the mice were housed in an air-conditioned room at 25 ± 1°C,and food and water were provided *ad libitum*.

### Tissue preparation

Mice were deeply anesthetized with 10% chloral hydrate (300 mg/kg, i.p.) and quickly transcardially perfused with 0.9% saline solution after different reperfusion durations of 0.5, 12, and 24 h, and 3, 7 and 14 days to measure time-dependent changes in protein expression. The brains were removed and cut at 3 (slice 1), 5 (slice 2), 7 (slice 3), 9 (slice 4), 11 (slice 5), and 13 (slice 6) mm posterior to the olfactory bulb into 2-mm thick coronal sections. To confirm the brain infarction, brain sections were stained with 2% 2.3.5-triphenyl tetrazolium chloride for 30 min at 37°C. Slices 2 and 3 were used for Western blot analysis, and slices 1-6 were fixed in 4% phosphate-buffered paraformaldehyde overnight, and then kept in 30% sucrose at 4°C until they sank, which were then coronally cut into 20 um thick sections using a freezing microtome (Jung Histocut, Model 820-II, Leica, Germany), mounted onto glass slides and stored at -20°C for immunofluorescence staining.

### BV2 cell culture and treatment

Mouse BV2 cells were cultured in 70% Dulbecco’s modified Eagle medium supplemented with 5% horse serum, 10% fetal bovine serum, 1% streptomycin, and 1% penicillin, in 6-well plates (Corning Incorporated, New York, NY, USA) at a density of 1×10^6^ cells per well. Cell cultures were incubated at 37°C in a humidified environment containing 5% CO_2_. PcDNA3.1-TOPK vector (Invitrogen, Carlsbad, CA, USA), TOPK siRNA1-4 (20 μmol/L, GenePharma) or a negative siRNA control was transfected into BV2 cells for 48 h using Lipofectamine RNAiMAX Transfection Reagent (Invitrogen, USA) according to the manufacturer’s protocol. The scrambled sequence as negative control is 5’-TTCTCCGAACGTGTCACGT3’. The siRNA1, siRNA2, siRNA3 and siRNA4 were designed to target the TOPK sequence as follows respectively: Pbk-Mus-727-747, 5’-GCTGCTTCATGGAGACATAAA-3’; Pbk-Mus- 1140-1160, 5’-GCACTAATGAGGATCCTAAAG-3’, Pbk-Mus-369-389, 5’-GGGT CAGCGTTTACCTAAT GA-3’, Pbk-Mus-662-682, 5’-GCTGTAATTCTCAG AG TTGCT-3’. After verified the TOPK expression using Western blot, TOPK siRNA4 was chosen to knockdown TOPK in the following BV2 cell and animal experiments. Three independent experiments were carried out.

### ELISA and RT-PCR

After transfection with pcDNA3.1-TOPK vector, TOPK siRNA or a negative siRNA control for 24 h, BV2 cells were treated with LPS (100ng/ml) and IFN-γ (20ng/ml) for another 24 h, then the cell supernatants were collected to detect the levels of tumor necrosis factor-α (TNF-α), interleukin-6 (IL-6) and interleukin-1β (IL-1β), transforming growth factor-β (TGF-β) using mice enzyme-linked immunosorbent assay kit (ELISA; Xinbosheng, Beijing, China) according to the instructions. The mRNA levels of SOCS3 and TGF-β were analyzed for the BV2 cells. An amount of 10^6^ cells were added to 1-mL Trizol (Invitrogen), and total RNA was extracted and 1-μg total RNA was taken for reverse transcription using the SuperScript III Reverse Transcriptase kit with oligo (dT) primer (Invitrogen). SOCS3 primers: 5′- AGA GCG GAT TCT ACT GGA GCG -3′ and 5′- CTG GAT GCG TAG GTT CTT GGT C -3′; TGF-β primers: 5′- ACA GAG AAG AAC TGC TGT GTG C -3′ and 5′- GGG TTG TGT TGG TTG TAG AGG -3′; 18S primers: 5′- GAC ACG GAC AGG ATT GAC AGA -3′ and 5′- GGA CAT CTA AGG GCA TCA CAG -3′; 18S was used as internal control.

### Intracerebroventricular injection of Letivirus, drug administration and measurement of infarct lesions

Lentivirus (10^9^ TU/ml) was prepared by GenePharma (Shanghai, China), and the overexpressed and knockdown sequence was subcloned to pLV5(EF-1aF/GFP&Puro) pLV3(H1/GFP&Puro) vectors respectively. The TOPK siRNA4 sequence is the most effective target and was packaged into lentivirus. Mice were randomly divided into six groups as follows: sham operation, MCAO + control lentivirus, MCAO + TOPK-overexpressing lentivirus, MCAO + TOPK-siRNA lentivirus, MCAO + TOPK-siRNA lentivirus + Romidepsin (FK228), MCAO + TOPK-siRNA-lentivirus + suberanilohydroxamic acid (SAHA) groups. For intracerebroventricular injection, the mice were placed in a small animal stereotaxic frame (David Kopf Instruments, Tujunga, CA, USA). The skull was exposed and a small-hole craniotomy performed based on predetermined stereotaxic coordinates (lateral 1.6 mm and antero-posterior 1 mm to the bregma, and horizontal 2 mm from the dura mater) [16]. 7 μL of a mixture of lentiviral particles (10^9^ TU/ml) containing TOPK overexpression, TOPK siRNA, or nontargeting control sequences were mixed with the cationic lipid polybrene (4 μg/μl, GenePharma), incubated at 37°C for 15 min, and manually injected into the right cerebral lateral ventricle slowly for ≥20 min using a glass micro-needle (Drummond Scientific Company, PA) at 7 days before 45min tMCAO. The selective HDAC1/2 inhibitor Romidepsin (FK228) and pan-HDAC inhibitor SAHA were dissolved in 10% DMSO and intraperitoneally injected at a dose of 0.5 mg/kg and 2.5 mg/kg respectively, immediately after tMCAO [17]. This injection was repeated twice per week and lasted for 2 weeks. Mice in the other four groups were intraperitoneally injected with 10% DMSO.

For histological assessment, the mice were deeply anesthetized with 10% chloral hydrate (300 mg/kg; i.p.) and perfused 14 days after ischemia-reperfusion, and the brain was immediately removed and cut into 2 mm slices before fixed in 4% phosphate-buffered paraformaldehyde overnight, and then kept in 30% sucrose at 4°C until they sank. Then tissues were sectioned at 20 um, mounted on glass slides, and six thin sections were selected from the each thick (2 mm) slice and stained with hematoxylin and eosin (HE) to determine the infarct lesions. Areas of both the ischemic and non-ischemic side were measured using Image J. Multiplying the result by the slice thickness (2 mm) gave an estimate of the volume in each slice. The total contralateral and ipsilateral hemisphere infarct volume was calculated by adding the volumes of contralateral and ipsilateral hemisphere in all slices respectively. The total brain tissue loss was calculated using the following formula: 100 × (the total contralateral hemisphere volume-total ipsilateral hemisphere vulume)/ total contralateral hemisphere volume. The analysis was performed by investigators blinded to the experimental groups.

### Rotarod test

For Rotarod testing, animals were placed on an accelerating rotating rod (4 to 40 rpm over 120s) and their latency to fall off the rod was recorded as previously reported [18]. Preoperative training was performed for 3 days with 3 daily trials, and those cannot run 300s would be excluded. The last three trials serve as a preoperative baseline. Postoperative testing was performed at 1, 3, 5, 7, 10 and 14 days after MCAO, for 3 trials per day, and the mean latency to fall was analyzed.

### Balance beam test

The experiment was performed on a horizontal rod (100 × 0.7 cm), approximately 40 cm above the surface. Prior to MCAO, mice were allowed to learn to traverse the beam and the maximum duration of the beam-walking test was set to 15 seconds to exclude those that did not cross the beam. The average footsteps that were used to traverse the beam were counted as 18. The testing was performed at 1, 3, 5, 7, 10 and 14 days after MCAO, for 3 trials per day, and the mean foot-slips were counted.

Locomotor skills were assessed using a 0-6 point rating scale, as described previously [19]. Numerical scores indicate the following performance ability: 0- Mice are unable to cross the beam or place the affected limb on the horizontal surface of the beam; 1- Mice place their affected limb on the beam surface but are unable to traverse; 2- Mice traverse the beam without placement of the affected limb; 3- Mice traverse the beam with placement at least once during Crossing; 4- Mice use their affected limb to aid less than 50% of the steps along the beam; 5- Mice use their affected limb to aid more than 50% of the steps along the beam; 6- Mice traverse the beam with 2 or fewer footslips.

### Western blotting

The ipsilateral brain tissue or BV2 cells were homogenized in lysis buffer (1% Triton X-100, 100 mM NaCl, and 50 mM Tris/HCl, pH 7.5), supplemented with phosphatase inhibitors (Sigma cocktail; Sigma, St. Louis, MO, USA) and protease inhibitors [leupeptin, aprotinin, pepstatin, and phenylmethylsulfonyl fluoride (PMSF)], followed by 3 rounds of sonication for 10 s. Lysates were spun at 12,000g for 30 min at 4°C. Protein concentration of supernatant was determined by bicinchoninic acid (BCA) assay (Thermo Scientific). 30 to 50 ug protein was separated by 8%, 10% or 12% sodium dodecyl sulfate polyacrylamide gel electrophoresis (SDS-PAGE) and electrophoretically transferred to polyvinylidene difluoride membranes (Bio-Rad; Hercules, CA). Membranes were blocked in blocking buffer (10% dried milk in Tris-buffered saline and Tween 20, (TBST) which contained 50 mM Tris, 150 mM NaCl, 0.05% Tween 20, pH adjusted to 7.4 with HCl) at 25°C for 1 hr and then incubated (4°C overnight) with the following primary antibodies (1:1000 dilution): rabbit anti-TOPK, rabbit anti-acetyl-histone H3 (Lys9/Lys14), rabbit anti-acetyl-histone H4 (Lys5), rabbit anti-histone H3, rabbit anti-HDAC1, rabbit anti-HDAC2 (Cell Signaling Technology, Waltham, MA, USA); rabbit anti-p-HDAC1 (phospho S421), rabbit anti-p-HDAC2 (phospho S394), rabbit anti-CD16, rabbit anti-iNOS, rabbit anti-CD206, rabbit anti-Arg1 (Abcam, Cambridge, MA, USA); rabbit anti-histone H4, and rabbit anti-β-actin (Santa Cruz Biotechnology, Santa Cruz, CA, USA). After rinsing 3x for 15 min each using TBST, the membranes were then incubated with species-specific horseradish peroxidase-conjugated secondary antibodies (Santa Cruz Biotechnology, 1:2000 dilution) for 60 min at room temperature. After three 10 min washes with TBST, membranes were developed with Millipore Luminata Classic Western horseradish peroxidase substrate for visualization on CL-XPosure autoradiography film (Thermo Scientific). Protein levels were quantified using AlphaEaseFC 4.0. And all of them were normalized to their respective β-actin levels.

### Immunofluorescence

The frozen sections were placed at room temperature for 0.5 hour after taken out from the -20°C and immersed in blocking solution (PBS containing 5% bovine serum albumin (BSA) and 0.3% Triton X-100) for 1.5 h. Sections were then incubated overnight at 4°C in blocking solution with primary antibody (1:100 dilution) (mouse anti-TOPK, Santa Cruz Biotechnology, Santa Cruz, CA; rat anti-CD16/CD32, BD Pharmingen, BD Biosciences; goat anti-CD206, R&D Systems; rabbit anti-p-HDAC1 S421, Abcam, Cambridge, MA; rabbit anti-p-HDAC2 S394, Abcam, Cambridge, MA; rabbit anti- ionized calcium binding adaptor molecule 1 (Iba1), Wako Chemicals, VA). After washing in PBS, sections were incubated with secondary antibodies in blocking solution (1:1000 dilution) (anti-mouse Alexa 488; anti-goat Alexa-594; anti-rat Alexa-594; anti-rabbit Alexa-594; anti-rabbit Alexa-488, Life Sciences, Paisley, UK) for 1.5 h. Sections were washed with PBS and coverslipped with Prolong antifade medium containing Dapi (Life Sciences, Paisley, UK). Images were captured with a fluorescence microscope.

### Co-immunoprecipitation (Co-IP)

For immunoprecipitation (IP), tissue homogenates (each containing 100 μg of proteins) were diluted 5-fold with RIPA buffer (Cell Signaling Technology, Waltham, MA, USA, 9806) containing 20 mM Tris-HCl (pH 7.5), 150 mM NaCl, 1% NP-40, 1% sodium deoxycholate, 2.5 mM sodium pyrophosphate, 1 μg/ml leupeptin and 1 mM each of EGTA, β-glycerophosphate, and Na3VO4. Samples were pre-incubated for 3 h with 30 μL Protein G Sepharose (Sigma, St. Lois, MO, P3296) and then centrifuged to remove any protein adhered nonspecifically to the Protein G Sepharose at 4°C. The supernatant was incubated with 3 μg of the indicated antibody (HDAC1, Abcam, Cambridge, MA; HDAC2, Abcam, Cambridge, MA), or species-relevant non-specific IgG (n.s. IgG, Cell Signaling Technology, Waltham, MA, USA) for an overnight incubation at 4°C. After the addition of 30 μL Protein G Sepharose, the mixture was incubated at 4°C for an additional 2 h. Samples were triple washed with IP buffer containing 10 mM Tris-Cl (pH 7.5), 150 mM NaCl, 2 mM EDTA and 0.5% Triton X-100, eluted with SDS-PAGE loading buffer, and boiled for 5 min. The levels of TOPK in the precipitate of the sham, MCAO 24 h, and MCAO 3 d groups (n=3), as well as the levels of TOPK (Santa Cruz Biotechnology, Santa Cruz, CA), HDAC1, and HDAC2 in brain tissue lysates were detected by Western blotting.

### Statistical analysis

All statistical analyses were performed using GraphPad Prism (Version 5.0, GraphPad Software, CA) as described in each figure legend. Data were expressed as the mean ± SEM and statistically analyzed with one-way ANOVA or two-way ANOVA followed by Tukey’s *post hoc* test. Data were considered significant when *p* < 0.05.

**Figure 1.**
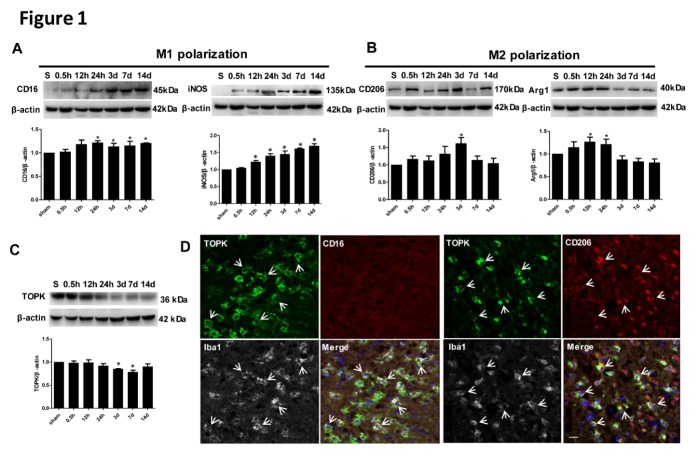
Time-dependent changes and localization of TOPK, M1, and M2 phenotype markers in the ipsilateral hemisphere of mouse brains after 45 min of ischemia with different reperfusion durations of 0.5 h, 12 h, 24 h, 3 days, 7 days, 14 days, and in the sham-operated group. (A-C) Western blot and corresponding quantitative analysis of protein expression of the M1 markers CD16 and iNOS, the M2 markers CD206 and Arg1, and TOPK. β-actin was used as the loading control. Values are expressed as the mean ± SEM. *p < 0.05 versus sham. N = 5 per group. S, sham group. (D) Representative immunofluorescence images stained for CD16 (red) or CD206 (red), TOPK (green), Iba1 (white) and DAPI (blue) in the ipsilateral cortex of mouse brain after 45 min ischemia and 3 days reperfusion. DAPI, nuclei. Scale bar = 20 µm. Statistics: Tukey’s honest significance test.

## RESULTS

### TOPK was expressed in parallel to and co-localized with M2 phenotype markers in brain tissues subjected to ischemia-reperfusion

To determine whether TOPK is related to microglia/macrophage M1/M2 polarization, the expression of M1 surface markers (CD16 and iNOS) and M2 surface markers (CD206 and Arg1) and their co-localization with TOPK were examined over time after cerebral ischemia-reperfusion. Western blot analysis showed the increased CD16 and iNOS starting at 24 h and 12 h, respectively, with high expression levels maintained for up to 14 days after ischemia (Fig. 1A). By contrast, the protein levels of the M2 markers CD206 and Arg1 increased at 24 h and 0.5 h, respectively, peaked at 3 days and 12 h, respectively, and began to decrease to base level at 7 days and 3 days after tMCAO (Fig. 1B). TOPK levels decreased progressively after 24 h, and reached the lowest level at 7 days (Fig. 1C), showing a similar pattern of expression as that of M2 surface markers. Immunofluorescence analysis showed that the M2 surface marker CD206 positive cells co-localized with TOPK and microglia marker Iba1, while we cannot see M1 phenotype marker CD16 positive cells in the TOPK positive microscopic field (Fig. 1D). The similar expression patterns of TOPK and the M2 surface maker after ischemia-reperfusion together with the co-localization of TOPK and Iba1 with CD206 after tMCAO suggest the possible involvement of TOPK in microglia/macrophage polarization after cerebral ischemia-reperfusion.

### TOPK overexpression primes microglia/macrophage toward the M2 phenotype in vitro

**Figure 2.**
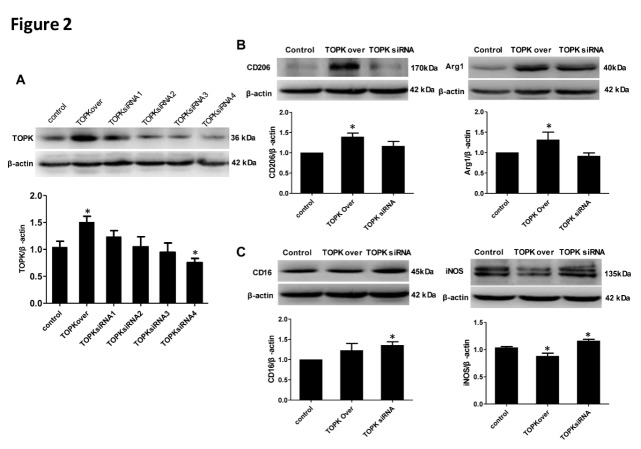
TOPK overexpression upregulated M2 marker protein expression in BV2 cells. (A) Western blot analysis verified the overexpression and knockdown TOPK after tranfection with TOPK overexpressed and siRNA1, 2, 3, 4 vector. (B-C) Western blot analysis of the expression of the M1 markers CD16 and iNOS, and the M2 markers CD206 and Arg1 at 48 h after TOPK overexpression and siRNA4 transfection, with quantification of the results shown below. β-actin was used as the loading control. Data are expressed as the mean ± SEM. *p < 0.05 versus control. N = 6. control=control vector. Statistics: Tukey’s honest significance test.

To investigate the effect of TOPK on M1/M2 polarization, the expression of M1 surface markers (CD16 and iNOS) and M2 markers (CD206 and Arg1) in BV2 cells was examined at 48 h after transfection with TOPK vector or treatment with TOPK siRNA1, 2, 3, 4. The overexpression and knockdown of TOPK in TOPK overexpressed and siRNA4 vectors transfected cells were verified by Western blotting (Fig. 2A, P < 0.05). Therefore, we chose the most effective TOPK siRNA4 to knockdown TOPK in the following experiments. TOPK overexpression significantly up-regulated the protein levels of M2 phenotype markers CD206 and Arg1 (Fig. 2B, *p* < 0.05), whereas it significantly down-regulated the protein levels of M1 phenotype markers iNOS and had no effect on that of CD16 (Fig. 2C). TOPK siRNA treatment up-regulated the expression of CD16 and iNOS (Fig. 2C), whereas had no effect on the levels of M2 markers. These results suggested that TOPK overexpression might promote microglia/macrophage polarization toward the M2 phenotype.

**Figure 3.**
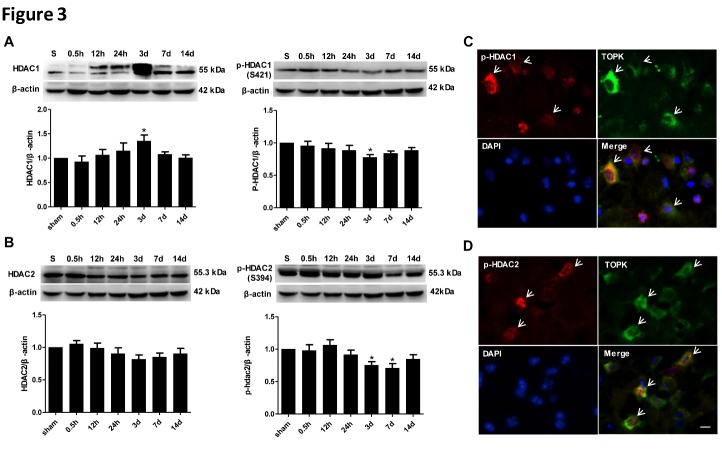
Changes in the expression of p-HDAC1, HDAC1, p-HDAC2, and HDAC2, and colocalization of p-HDAC1 and p-HDAC2 with TOPK following cerebral ischemia-reperfusion. (A and B) Changes in the protein expression of HDAC1, p-HDAC1, HDAC2, and p-HDAC2 in the ipsilateral hemisphere of mouse brains after 45 min ischemia with different reperfusion durations of 0.5 h, 12 h, 24 h, 3 days, 7 days, and 14 days, and in the sham-operated group. Quantification of the results is shown below. β-actin was used as the loading control. Data are expressed as the mean ± SEM. *P < 0.05 versus control. N = 5. (C and D) Representative double-staining immunofluorescence of p-HDAC1/TOPK and p-HDAC2/TOPK in the ischemic cortex of ipsilateral brain at 3 days after ischemia-reperfusion. Scale bar: 20 μm. N = 5. *p < 0.05 versus sham. Statistics: Tukey’s honest significance test.

### TOPK expression and HDAC1/2 phosphorylation after cerebral ischemia-reperfusion

To investigate whether the effect of TOPK on microglia/macrophage polarization is mediated by the regulation of HDAC1/2, the expression of HDAC1, p-HDAC1, HDAC2, and p-HDAC2, and their co-localization with TOPK were examined in brain tissues subjected to cerebral ischemia-reperfusion. Western blot analysis showed an increase in the levels of total HDAC1 at 3 days concomitant with the down-regulation of phosphorylated HDAC1. Total HDAC2 tend to decrease at 3 and 7 days concomitant with a marked down-regulation of phosphorylated HDAC2. P-HDAC1 and p-HDAC2, but not total HDAC1 or HDAC2, showed a similar pattern of expression to that of TOPK (Fig. 3A and B). In addition, TOPK positive cells co-localized with p-HDAC1 and p-HDAC2, as demonstrated by immunofluorescence analysis (Fig. 3C and D).

### TOPK binds to HDAC1 and HDAC2 after cerebral ischemia-reperfusion

**Figure 4.**
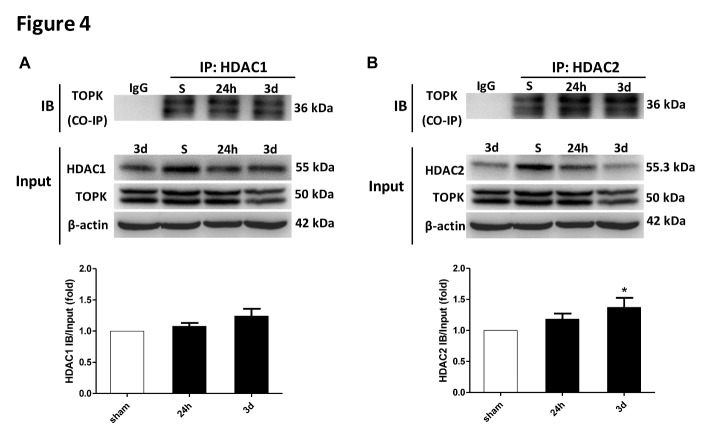
TOPK binds to HDAC1 and HDAC2 in brain tissues under normal conditions and following brain ischemia-reperfusion. (A) Immunoprecipitation was performed with a TOPK antibody, followed by immunoblotting with HDAC1 antibody or TOPK antibody (top panel). Bands of 55 and 50 kDa represent HDAC1 and TOPK, respectively, with quantification of the results (bottom panel). (B) Immunoprecipitation was performed with TOPK antibody, followed by immunoblotting with HDAC2 antibody or TOPK antibody (top panel). Bands of 55.3 and 50 kDa represent HDAC2 and TOPK, respectively, with quantification of the results (bottom panel). Data are expressed as the mean ± SEM. *p < 0.05 versus sham, N = 3. Statistics: Tukey’s honest significance test.

To verify the direct interaction between TOPK and HDAC1/HDAC2 *in vivo*, we performed Co-IP analyses. The results of Co-IP showed binding of TOPK to HDAC1 and HDAC2 in brain tissues both under normal conditions and following brain ischemia-reperfusion, with increased binding over time and a significant interaction detected at 3 days for HDAC2 (Fig. 4A and B). This timing coincided with the significant reduction in the phosphorylation of HDAC1 and HDAC2 (Fig. 3A and B).

### TOPK overexpression promotes HDAC1/HDAC2 phosphorylation and Histone 3 and Histone 4 acetylation in BV2 cells

**Figure 5.**
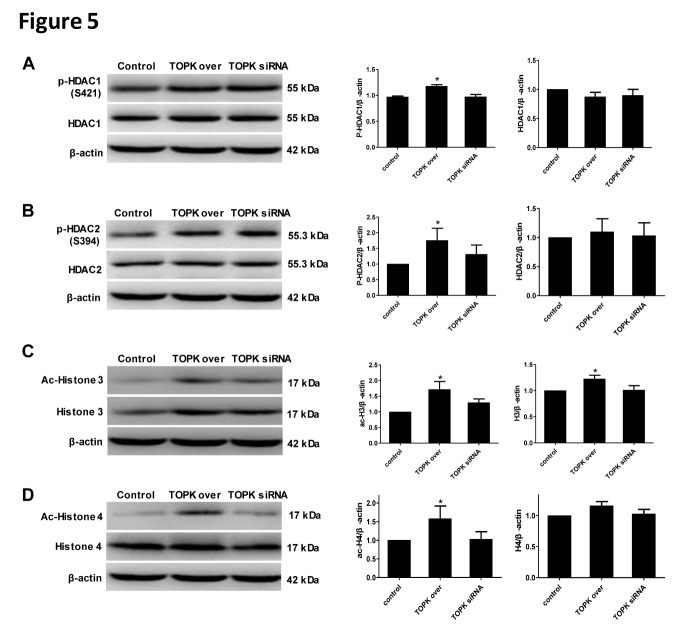
Effect of TOPK overexpression and TOPK siRNA on HDAC1 and HDAC2 phosphorylation, and Histone 3 and Histone 4 acetylation in BV2 cells. (A-D) The expression of p-HDAC1/HDAC1, p-HDAC2/HDAC2, ac-H3/H3, and ac-H4/H4 in BV2 cells was determined by western blotting after transfection with TOPK overexpressing and TOPK siRNA vector for 48 h. Data are expressed as the mean ± SEM. * *p* < 0.05 versus control, N = 6. Statistics: Tukey’s honest significance test.

To further investigate whether the interaction between TOPK and HDAC1/HDAC2 affected on their post-transcriptional modifications or protein expression in microglia/macrophage, the levels of phosphorylated and total HDAC1/HDAC2 were examined in BV2 cells transfected with TOPK overexpressing or TOPK siRNA vector. Western blot analysis showed that p-HDAC1, p-HDAC2, ac-histone 3, and ac-histone 4 were significantly up-regulated in response to TOPK overexpression, with no changes in total HDAC1, total HDAC2, and total histone 4 (Fig. 5A-D; *p* < 0.05). TOPK knockdown had no effect on the levels of phosphorylated and total HDAC1/2 or ac-histone 3 and ac-histone 4. These results indicated that TOPK overexpression resulted in the phosphorylation of HDAC1 and HDAC2, which might inactivate them and promote the acetylation of Histone 3 and Histone 4.

### TOPK overexpression and the HDAC1/HDAC2 inhibitor FK228 both drive microglia/macrophage polarization towards the M2 phenotype, alleviate brain injury and ameliorate neurological functions deficits in mice after tMCAO

**Figure 6.**
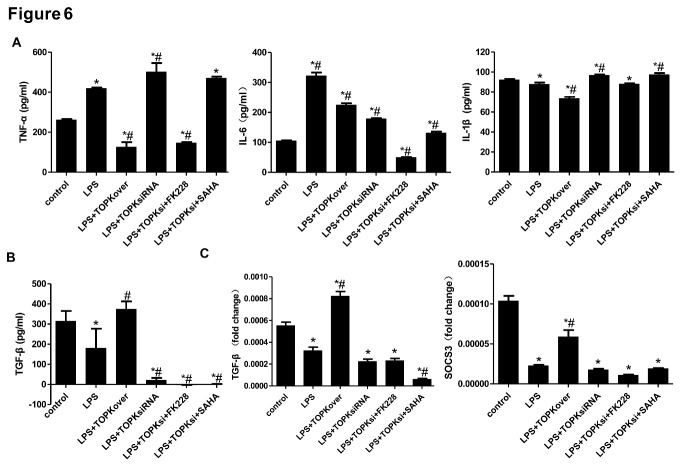
TOPK influences inflammatory response in BV2 cells following LPS plus IFN-γ stimulation. (A) ELISA analysis of TNF-α, IL-6 and IL-1β levels in the culture medium of LPS (100 ng/ml) plus IFN-γ (20 ng/ml)-induced BV2 cells for 24 h. (B) ELISA analysis of TGF-β level in the culture medium of LPS (100 ng/ml) plus IFN-γ (20 ng/ml)-induced BV2 cells for 24 h. (C) Determination of mRNA expression of M2 phenotype, TGF-β and SOCS3 by RT-PCR. LPS, lipopolysaccharide. Data are expressed as the mean ± SEM. N=6. *p < 0.05 versus control. ^#^*p* < 0.05 versus LPS. Statistics: Tukey’s honest significance test.

To examine whether the up-regulation of M2 surface markers by TOPK overexpression was mediated by the inhibition of HDAC1/HDAC2 after ischemia-reperfusion *in vitro* and *in vivo*, TOPK-overexpressing vector and small interfering RNA was transfected to BV2 cells, and TOPK-overexpressing and small interfering RNA lentivirus was delivered by intracerebroventricular injection at 7 days before tMCAO as well. The ELISA analysis showed that TOPK overexpression significantly reduced LPS plus IFN-γ-induced release of TNF-α, IL-6 and IL-1β, the inflammatory factor of M1 phenotype, and up-regulated that of M2 phenotype, TGF-β (Fig. 6A and B; p < 0.05). On the contrary, TOPK siRNA increased the expression of M1 phenotype inflammatory factor TNF-α and IL-1β induced by LPS while reduced that of M2 phenotype TGF-β. Moreover, the selective HDAC1/ HDAC2 inhibitor FK228 but not the pan-HDAC inhibitor SAHA reversed the up-regulation of M1 phenotype inflammatory factor TNF-α induced by TOPK siRNA. The RT-PCR analysis demonstrated that LPS plus IFN-γ decreased the mRNA expression of M2 phenotype, TGF-β and SOCS3, which were up-regulated by TOPK overexpression (Fig. 6C; *p* < 0.05), while TOPK siRNA alone or plus FK228/SAHA did not influence their levels.

**Figure 7.**
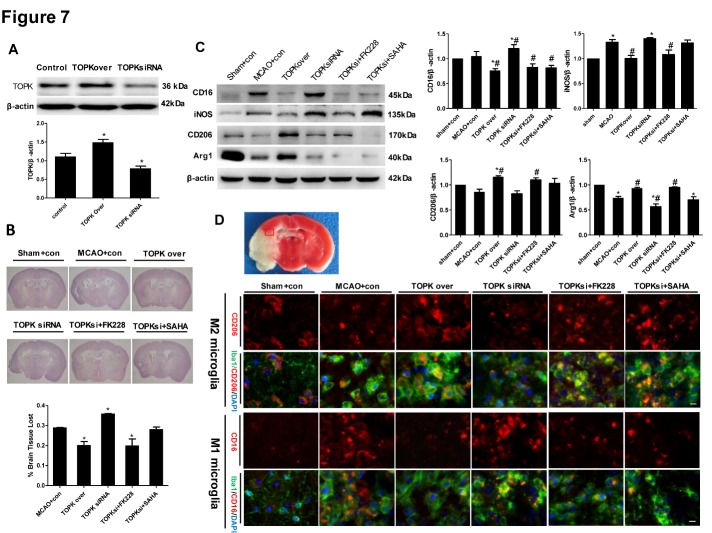
Effects of TOPK overexpression, TOPK siRNA, the HDAC1/2 specific inhibitor FK228, and the broad-spectrum HDACi SAHA on microglia/macrophage M1/M2 polarization and cerebral injury in mice following 45 min ischemia/14 days reperfusion. (A) The western blotting verified the overexpression and knockdown of TOPK in ipsilateral brain tissue of mice intracerebroventricularly injected with TOPK overexpressing and siRNA4 letivirus. (B) Representative images of HE-stained sections (upper panels) and assessment of cerebral atrophy (lower panel). (C) Western blot (left) and corresponding quantitative analysis (right) of the expression of M1 markers (CD16 and iNOS) and M2 markers (CD206 and Arg1) in the ipsilateral brain of ischemic mice. *p < 0.05 versus sham, ^#^*p* < 0.05 versus MCAO. (D) Immunofluorescence images stained for the M2 marker CD206 (red, first row) or M1 marker CD16 (red, third row), the microglia/macrophage marker Iba1 (green) and DAPI (blue), which showed the changes in CD206 and CD16 positive microglia/macrophage in the ipsilesional brain at 14 days following tMCAO (vehicle, TOPK overexpression, TOPK siRNA, TOPK siRNA+FK228 and TOPK siRNA +SAHA groups) or sham surgery (sham group). Images were taken from the red-boxed area in the brain slice of TTC staining. Scale bar: 20 μm. con, control letivirus. N=6. Statistics: Tukey’s honest significance test.

The results of Western blotting verified the overexpression and knockdown of TOPK in ipsilateral brain tissue of mice intracerebroventricularly injected with TOPK overexpressing and TOPK siRNA letivirus (Fig. 7A; *p* < 0.05). TOPK overexpression markedly decreased brain tissue loss at 14 days after MCAO compared with that in the MCAO group (Fig. 7B; *p* < 0.05). Consistently, TOPK siRNA increased brain tissue loss (*p* < 0.05), and this effect was suppressed by the selective HDAC1/HDAC2 inhibitor FK228 but not by the broad-acting HDAC inhibitor SAHA (*p* < 0.05). Western blot analysis showed that TOPK overexpression significantly downregulated the M1 surface markers CD16 and iNOS and significantly upregulated the M2 surface markers CD206 and Arg1 compared with the levels in the MCAO group (Fig. 7C; *p* < 0.05). By contrast, TOPK siRNA up-regulated CD16 and down-regulated Arg1 significantly compared with the MCAO group, and this effect was reversed by the selective HDAC1/HDAC2 inhibitor FK228 and the broad-acting HDAC inhibitor SAHA (Fig. 7C; *p* < 0.05). These results were confirmed by double staining for the microglia/macrophage marker Iba1 and CD206 or CD16 in the ipsilateral brain section at 14 days after different treatments. The results of immunofluorescence analysis showed that the changes in CD206 and CD16 positive microglia/macrophage were similar to the changes in M2 and M1 surface marker expression in the western blot analysis, respectively (Fig. 7D).

**Figure 8.**
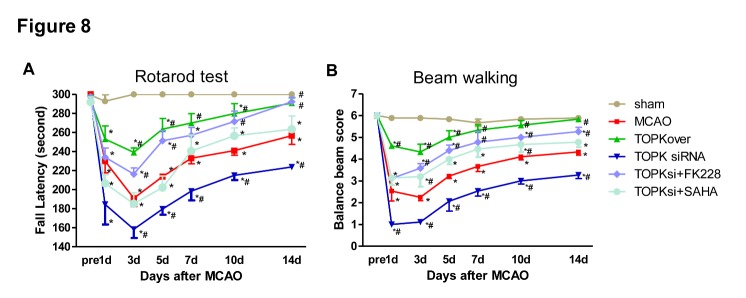
Effects of TOPK overexpression, TOPK siRNA, the HDAC1/2 specific inhibitor FK228, and the broad-spectrum HDACi SAHA on the sensorimotor functions after MCAO. (A and B) Rotarod test and Balance beam test showed the changes in sensorimotor functions up to 14 days following tMCAO (vehicle, TOPK overexpression, TOPK siRNA, TOPK siRNA+FK228 and TOPK siRNA+SAHA groups) or sham surgery (sham group). Shown are the mean ± SEM. *p < 0.05 versus sham, ^#^*p* < 0.05 versus MCAO. N=6. Statistics: Tukey’s honest significance test.

Next, we evaluated the effect of TOPK and HDAC inhibitor on functional outcomes after MCAO. The Rotarod test and Balance Beam test both exhibited significant improvement in sensorimotor deficits lasting for at least 14 days following MCAO in TOPK overexpression groups, whereas TOPK siRNA significantly aggravated the sensorimotor deficits, which were completely reversed by FK228 (Fig. 8A and B; *p* < 0.05).

These findings indirectly suggested that TOPK promoted microglia/macrophage polarization towards the M2 phenotype by inhibiting HDAC1/HDAC2 activity, thus exerting neuroprotective effects against cerebral ischemia-reperfusion.

## DISCUSSION

Cumulative studies indicate that the dual role of activated microglia/macrophages depends on the transition between the “classically activated” pro-inflammatory M1 phenotype and the “alternatively activated” neuroprotective M2 phenotype [20-25]. This suggests that regulating the phenotype transition may be a promising target for ischemic stroke therapy. Several studies report the involvement of TOPK in inflammatory responses [26, 27]. However, the function of TOPK in microglia/macrophage M1/M2 polarization following brain ischemic injury remains unknown. In the current study, we showed that TOPK expression after cerebral ischemia-reperfusion followed a similar pattern to that of the M2 surface markers CD206 and Arg1. Furthermore, TOPK co-localized with CD206 in the ischemic region. Our *in vitro* studies demonstrated that TOPK overexpression significantly up-regulated CD206 and Arg1 in BV2 cells. These data highlighted the critical importance of TOPK in driving microglia/macrophage polarization towards the M2 phenotype following cerebral ischemia-reperfusion injury, and suggested that TOPK might be a potential therapeutic target in ischemic stroke. However, TOPK overexpression also had values both as a predictive biomarker and prognostic factor in lung cancer, ovarian cancer and skin inflammation, which made TOPK silencing or inhibition a potential therapeutic target in these diseases [28-30]. Above all, we have to think over carefully before we translate those scientific achievements into clinical application, and the TOPK activity should been brought under cautious control in a dose and time dependent manner.

The mechanism underlying the effect of TOPK on promoting microglia/macrophage polarization towards the M2 phenotype remains unclear. An increasing number of studies have agreed on the role of HDACi in regulating inflammatory responses in microglia/macrophages. Broad-spectrum valproic acid and sodium butyrate significantly enhanced the release of PGE2, PGD2, 8-iso-PGF2α, TNF-α, and IL-1β in 24 h LPS-activated primary microglia [31], while TSA strongly suppressed the expression of both M1 markers (IL-1b, IL-6, and TNF-α) and M2 markers (Arg 1 and Fizz1) induced in LPS or IL-4-treated primary microglia, respectively [7]. In addition, TSA led to a transition from the typical macrophage pancake-like shape to an elongated morphology, and this correlated with a mixed M1/M2 profile of cytokine and chemokine secretion [32]. Class I HDACs were widely expressed in astrocytes, distal microglial processes, and primary glial cells [33, 34], and their selective inhibition could have a protective effect against ischemia-reperfusion injury [35]. Moreover, HDAC1 and HDAC2 were most abundantly expressed by mouse primary microglia and most significantly changed by LPS stimulation *in vitro* and *in vivo* [7]. HDAC1 and HDAC2 displayed both overlapping and non-redundant functions in proliferation [36]. Based on these results, we investigated whether the involvement of TOPK in microglia/macrophage M1/M2 polarization was related to HDAC1/HDAC2. Our data showed that the time-dependent changes in p-HDAC1 and p-HDAC2 expression occurred in parallel to that of TOPK after ischemia-reperfusion. Moreover, TOPK co-localized with p-HDAC1 and p-HDAC2 by immunofluorescence, which was confirmed by the Co-IP analysis showing that TOPK binds to HDAC1 and HDAC2 in brain tissues both under normal conditions and following cerebral ischemia-reperfusion. The results of *in vitro* studies further confirmed the effect of TOPK on HDAC activity by showing that TOPK overexpression significantly up-regulated p-HDAC1 and p-HDAC2, resulting in an increase in the acetylation of histones H3 and H4 in BV2 cells. These data suggested that TOPK phosphorylated HDAC1 and HDAC2, which then acetylated histones and promoted M2 surface marker expression. This idea was further supported by the fact that TOPK overexpression increased M2 surface marker expression *in vitro* and *in vivo*, reduced cerebral atrophy and improved the neurological functions significantly following cerebral ischemia-reperfusion. Conversely, TOPK siRNA down-regulated M2 marker, aggravated cerebral atrophy, as well as the sensorimotor functions, which was completely reversed by the selective HDAC1/HDAC2 specific inhibitor FK228. Although the pan-HDACi SAHA also increased M2 surface expression similar to FK228, it did not attenuate cerebral atrophy or alleviate the neurological deficits in this study. SAHA was the first US Food and Drug Administration approved HDAC inhibitor to be used in the treatment of cutaneous T-cell lymphoma [37]; however, the phase I and II studies also showed numerous side effects such as bone marrow depression and disordered clotting among others [38]. Considering the wide distribution and the crucial roles of HDACs in the regulation of chromatin structure and post-translational modification of numerous key proteins, this result was understandable.

In summary, our study demonstrated the involvement of TOPK in microglia/macrophage M1/M2 polarization following cerebral ischemia-reperfusion injury. In the subacute phase, the decrease in TOPK levels was accompanied by the down-regulation of M2 surface markers and phosphorylation of HDAC1/HDAC2 following cerebral ischemia-reperfusion *in vivo*. The co-localization and binding of TOPK to M2 surface markers or HDAC1/HDAC2 supported their interaction. TOPK may drive microglia/macrophage polarization towards the M2 phenotype through the phosphorylation of HDAC1 and HDAC2 was confirmed by TOPK overexpression, TOPK siRNA, and selective HDAC1/2 inhibitor FK228 treatment *in vitro* and *in vivo*. Our previous investigation showed that TOPK attenuate the oxidative stress during the acute stage following stroke through activating Akt pathway, and the present results indicate that TOPK may ameliorate brain ischemia-reperfusion injury during the recovery stage by driving a shift of microglia/ macrophages towards the M2 phenotype through the phosphorylation of HDAC1 and HDAC2, suggesting TOPK functions through different mechanism at different stage following stroke, which could become a promising target for new drug development in the treatment of ischemic stroke.
